# Exogenous IFN-α delivered as an intranasal adjunct to systemic vaccination directs vaccine-specific T-cell responses to the upper respiratory tract

**DOI:** 10.3389/fimmu.2026.1924673

**Published:** 2026-09-18

**Authors:** Rupsha Fraser, Jürgen Schwarze, David H. Dockrell

**Affiliations:** 1 Centre for Inflammation Research, Institute for Regeneration and Repair, The University of Edinburgh, Edinburgh, United Kingdom; 2 Child Life and Health, Royal Hospital for Children and Young People, The University of Edinburgh, Edinburgh, United Kingdom

**Keywords:** interferon-alpha (IFN-α), mucosal immunity, nasal immunomodulation, respiratory infections, systemic vaccination, upper respiratory tract (URT), nasal mucosa

## Abstract

Respiratory infectious diseases remain a major global health burden, sustained by ongoing community transmission. Licensed respiratory vaccines are predominantly systemically administered, which elicit vaccine-induced antigen-specific circulating immunity, but fail to protect the primary site of initial infection and onward transmission, the upper respiratory tract (URT). The URT is a highly compartmentalised anatomical site that is not directly accessed by systemic vaccination. Consequently, conventional systemic respiratory vaccines do not reliably prevent acquisition of infection or its onward transmission, allowing continued circulation and evolutionary pressure on respiratory pathogens, whilst vulnerable populations remain disproportionately affected. To overcome this critical limitation of respiratory vaccinology, we present a novel intervention that combines systemic vaccination with targeted non-antigenic immunomodulatory stimulation of the nasal mucosa to generate vaccine antigen-specific immunity in the URT. This approach may offer an easily deployable route for use across diverse vaccination programmes to interrupt respiratory infection and transmission chains at a population scale. Here, using SARS-CoV-2 vaccination as a test case, we demonstrate for the first time that intranasal exogenous IFN-α, when coadministered as an adjunct to systemic vaccination, provides local immune instruction without reliance on intranasal antigen delivery, and directs vaccine-specific T-cell responses to the URT.

## Introduction

Community-acquired respiratory infections are collectively recognised as a leading healthcare and economic burden with substantial morbidity and mortality rates worldwide, especially at the extremes of age and in immunocompromised individuals. Over the past two decades, respiratory viruses have driven nine epidemics and pandemics (including the COVID-19 pandemic); and their ongoing emergence, persistence and evolution continue to threaten global health security. Vaccines against respiratory viruses predominantly have systemic administration routes, which generate vaccine-specific adaptive immunity in the circulation. However, the upper respiratory tract (URT), where host cells are initially targeted by respiratory viruses for initial replication and onward transmission (1, 2), is a compartmentalised anatomical site that is not directly accessed by systemic vaccination (3, 4). Thus, systemic respiratory vaccines are poor inducers of antigen-specific responses at the URT, and do not effectively block acquisition of infection and onward community transmission (5–7). Prevention of infection and transmission therefore remains a major unmet need in respiratory vaccinology.

To address this critical gap, we present an innovative intervention using SARS-CoV-2 vaccination as a test case, and demonstrate in this proof-of-concept report that targeted immunomodulatory conditioning of the nasal mucosa with exogenous interferon-alpha (IFN-α), when used as an adjunct to systemic vaccination, enables localisation of vaccine-specific T-cell responses to the URT. This is a previously undescribed adjunct intranasal immunomodulatory strategy for systemic respiratory vaccination, which uses a non-antigenic nasal innate immune signal to direct vaccine-induced immunity to the URT without reliance on local intranasal antigen delivery.

Whilst intranasal vaccination platforms can elicit robust vaccine-specific URT responses in experimental animals, they have to date largely failed to meet efficacy criteria for success in humans, with the exception of AstraZeneca’s paediatric intranasal live attenuated influenza vaccine (marketed as Fluenz® in Europe, FluMist® in North America). A major barrier is nasal mucociliary clearance, which limits absorption and bioavailability, as discussed in Fraser et al., 2023 (8). In contrast, our approach circumvents the longstanding technical challenges associated with intranasal antigen delivery, by using innate immune modulation to direct vaccine-induced adaptive responses to the URT mucosa. Alternative approaches for inducing vaccine-specific URT responses involve systemic priming followed by intranasal antigen boosting (9), which face similar challenges to intranasal vaccination, and temporally separated ‘prime and pull’ strategies that require delayed mucosal chemotactic stimulation after systemic priming (10, 11). These approaches are conceptually and operationally distinct from the strategy we introduce here, which excludes intranasal antigen administration, and uses simultaneous nasal immune conditioning and systemic vaccination, rather than delayed nasal mucosal stimulation after systemic priming.

It is anticipated that by directing vaccine-induced immunity to the site of pathogen entry at the URT, our innovation offers a compelling strategy with the potential to interrupt infection and transmission chains, and slow down viral evolution and variant emergence, including in the context of pandemic preparedness and public health emergencies.

## Materials and methods

### Study design

The goal of the study was to demonstrate the utility of intranasal IFN-α, when coadministered as an adjunct to systemic SARS-CoV-2 vaccination, in generating vaccine-specific immunity at the URT, the main port of viral entry and exit. Following treatments, T-cell responses in the URT were assessed to determine vaccine-reactivity. For this purpose, a non-transgenic mouse strain was used, as susceptibility to SARS-CoV-2 infection was not required for the core question addressed here.

### Mice

Murine studies were conducted under Home Office project licence number PP4544912, and in accordance with the UK Animals Act. A protocol was prepared for regulatory/ethical approval, but was not registered in a public registry. Animals were housed in pathogen-free conditions in ventilated cages with standard environmental enrichment, food and water available ad libitum, at a constant temperature and humidity, and a fixed light-dark lighting cycle. Animals were acclimatised for 14 days before experimental procedures. Treatments were administered at the same time of day to avoid circadian rhythm effects, and animals were cohoused prior to commencement of and during experiments to normalise microbiome effects. Animals were also monitored closely for potential side effects of intranasal IFN-α administration, such as localised haemorrhage, anaphylaxis or >15% weight loss.

### Mouse experiments

Animals were randomly assigned to experimental groups of equal size (n=5 per experimental group; n=15 total per experiment for all experiments), in which the individual mouse was the experimental unit. Female C57BL/6 mice (9–12 weeks) were randomised to groups and anaesthetised with isoflurane. Animals received intraperitoneal vaccination with 6 × 10^9^ viral particles of Vaxzevria AZD1222 (AstraZeneca SARS-CoV-2 vaccine; 61µl) on both days 0 and 21, with or without intranasal coadministration of 4 × 10^5^ U recombinant mouse IFN-α (4 × 10^5^ U IFN-α/10µl PBS; 5µl/nostril, 10µl total per animal) (BioLegend, London, UK), and were euthanised on day 42 together with naïve controls (**Supplementary Figure 1A**) via cervical dislocation in accordance with Schedule 1 of the UK Animals (Scientific Procedures) Act 1986, with death confirmed by exsanguination. AZD1222 was obtained under a materials transfer agreement between the University of Oxford/AstraZeneca and the University of Edinburgh. In an additional control experiment, mice were given 4 × 10^5^ U intranasal IFN-α alone (without concomitant systemic vaccination) on days 0 or 19, and euthanised on day 21 (i.e. 21 days or 48 hours post-treatment, respectively), together with naïve controls (**Supplementary Figure 1B**), using the same Schedule 1 procedure (cervical dislocation and confirmation of death by exsanguination). Following euthanisation in all experiments, URT tissues from each animal (nasopharynx-associated lymphoid tissue, nasal turbinates, cervical lymph nodes) were harvested, single cell suspensions prepared for *ex vivo* stimulation with SARS-CoV-2 Spike glycoprotein peptide pools (containing the surface antigen from AZD1222), and the single cell suspensions obtained from all three URT anatomical sublocations combined for each animal. Briefly, URT tissues from the different anatomical sublocations were obtained aseptically as previously described (12, 13), washed individually in HBSS buffer (Gibco, Paisley, UK), and pooled for each animal. The tissues were mechanically fragmented with sterile scalpels and scissors in phenol red-free RPMI 1640 containing 50 µg/ml pen/strep and 2.5 µg/ml amphotericin (Gibco, Paisley, UK), followed by enzymatic digestion with 3mg/ml collagenase D (Roche, Hertfordshire, UK) and 30μg/ml DNase I (Thermo Fisher Scientific, Dorset, UK) for 30 minutes at 37°C with constant shaking, and processed in a GentleMACS tissue dissociator (Miltenyi Biotec, Surrey, UK). Red blood cells in the homogenates were subsequently lysed with ACK lysis buffer (Gibco, Paisley, UK), and the suspensions then washed, centrifuged at 500 x *g* for 10 minutes at 4°C, and filtered through a 70-μm cell strainer.

### *Ex vivo* cell stimulation

Single cell suspensions were resuspended in phenol red-free RPMI 1640 supplemented with 10% FBS, 2mM L-glutamine, 1% MEM NEAA, 1% Na-pyruvate, 0.1% 2-betamercaptoethanol, 50 µg/ml pen/strep and 2.5 µg/ml amphotericin (Gibco, Paisley, UK). Cells were stimulated with SARS-CoV-2 Spike peptide pools (ImmunoServ, Cardiff, UK) at a final concentration of 1μg/ml per test for 18 hours at 37°C in a 5% CO_2_ humidified incubator, with 1 × 10^6^ isolated cells cultured per stimulation test. 5µg/ml brefeldin A and 2µM monensin (BioLegend, London, UK) were added after 4 hours to block extracellular IFN-γ protein transport, to enable flow cytometric detection of increased intracellular IFN-γ expression in CD3^+^CD4^+^ or CD3^+^CD8^+^ T-cells for determining vaccine reactivity. In addition, cells from each group were pooled for stimulation with a non-specific cell stimulation cocktail (eBioscience, Altrincham, UK) as a positive control for 18 hours, again with addition of 5µg/ml brefeldin A and 2µM monensin after 4 hours.

### Flow cytometry

Increased intracellular IFN-γ expression by CD3^+^CD4^+^ or CD3^+^CD8^+^ T-cells was assessed by flow cytometry to determine specific reactivity to SARS-CoV-2 Spike peptide pools (i.e. vaccine-reactivity). Following stimulation, cells were washed twice with PBS and centrifuged at 350 x *g* for 5 minutes at 4°C, stained with a surface marker antibody cocktail containing CD45-AF700, CD3-FITC, CD4-BV605, CD8a-PerCP/Cy5.5 antibodies (BioLegend, London, UK) for 45 minutes in the dark at RT. An additional aliquot of cells taken from a pool of all samples was kept aside as a negative control without subsequent IFN-γ staining, referred to as the fluorescence-minus-one (FMO) negative control to determine the background fluorescence threshold (i.e. by using an antibody cocktail containing all the antibodies used minus the IFN-γ-APC antibody, to differentiate true IFN-γ-positive events from nonspecific signals). All cells (except the pooled sample serving as the FMO negative control) were washed with PBS and centrifuged at 350 x *g* for 5 minutes at 4°C, fixed and permeabilised using the Cyto-Fast™ Fix/Perm kit according to manufacturer’s instructions (BioLegend, London, UK), followed by intracellular staining using an IFN-γ-APC antibody (BioLegend, London, UK) for 45 minutes in the dark at RT. Cells were washed twice with PBS and centrifuged at 350 x *g* for 5 minutes at 4°C, and resuspended in FACS buffer for flow cytometric analyses. Sample acquisition was performed on a BD LSR Fortessa 5L flow cytometer (BD Life Sciences, Berkshire, UK). At least 50,000 cells were acquired and analysed per sample, except for one sample from which 37,000 cells were acquired. Data were analysed in FlowJo software version 10.10.1 (BD Life Sciences, OR, USA). Vaccine-specific activated T-cells were defined as CD45^+^CD3^+^CD4^+^IFN-γ^+^ or CD45^+^CD3^+^CD8^+^IFN-γ^+^ cells, identified using sequential gating on cell size (SSC-A vs. FSC-A), singlets (FSC-H vs. FSC-A), and viability (LIVE/DEAD-negative).

### Experimental group size power calculation

Using a two-sided two-sample t-test with a significance level of 0.017, the effect size (δ) was calculated as δ = (µ_1_ - µ_2_)/σ based on the frequency of CD4^+^IFN-γ^+^ T-cells. δ = 8.608 was calculated from µ_1_ = 37.340; μ_2_ = 0.156; σ = pooled SD = 4.320. For the minimum detectable difference, with 80% power, an experimental group size of at least n=3 is required.

### Statistical analysis

No animals or data points were excluded from the analyses. Following assessment of normality using the Shapiro-Wilk test, differences in the frequencies of CD4^+^IFN-γ^+^ and CD8^+^IFN-γ^+^ T-cells amongst experimental groups were analysed using one-way ANOVA with Tukey’s *post-hoc* multiple comparisons test for parametric data, or the Kruskal-Wallis test with Dunn’s *post-hoc* multiple comparisons test for non-parametric data, using GraphPad Prism (GraphPad Software, San Diego, CA, USA). *P* < 0.05 was considered statistically significant.

## Results

### Intranasal IFN-α and systemic AZD1222 coadministration induced vaccine-specific T-cell responses in the URT

To evaluate the induction of antigen-specific T-cell responses in the URT, intracellular cytokine staining and flow cytometric analyses were performed following *ex vivo* stimulation with SARS-CoV-2 Spike peptide pools. Vaccine-specific T-cells were defined as CD45^+^CD3^+^CD4^+^IFN-γ^+^ or CD45^+^CD3^+^CD8^+^IFN-γ^+^ cells, identified using sequential gating on cell size (SSC-A vs. FSC-A), singlets (FSC-H vs. FSC-A), and viability (LIVE/DEAD-negative). FlowJo-based quantification revealed a marked induction of IFN-γ-producing CD4^+^ and CD8^+^ URT T-cells following coadministration of intranasal IFN-α with systemic AZD1222 vaccination in C57BL/6 mice. The percentage of IFN-γ^+^CD4^+^ or IFN-γ^+^CD8^+^ T-cells in peptide-stimulated samples was calculated after subtracting background signal from the FMO negative control, with the positive control (cells stimulated with a non-specific cell stimulation cocktail) confirming detection of IFN-γ-positive cells above the threshold defined by the FMO negative control. CD4^+^IFN-γ^+^ and CD8^+^IFN-γ^+^ T-cell frequencies were higher in the intranasal IFN-α + systemic AZD1222 group (37.34% and 47.38%, respectively) compared to the systemic AZD1222 alone group (0.16% and 1.8%, respectively) and the untreated group (0.01% and 0.096%, respectively). The Shapiro-Wilk normality test determined that the CD4^+^IFN-γ^+^, but not CD8^+^IFN-γ^+^, T-cell frequencies were normally distributed. The datasets were therefore analysed using one-way ANOVA (parametric) and the Kruskal-Wallis test (non-parametric), respectively, with significant differences amongst the experimental groups for both CD4^+^IFN-γ^+^ and CD8^+^IFN-γ^+^ T-cells (*P* < 0.0001). *Post-hoc* multiple comparison tests also identified significant differences in CD4^+^IFN-γ^+^ T-cell frequencies between the intranasal IFN-α + systemic AZD1222 group and both the systemic AZD1222 alone and untreated groups (Tukey’s, *P* < 0.0001 for both comparisons), and in CD8^+^IFN-γ^+^ T-cell frequencies between the intranasal IFN-α + systemic AZD1222 and untreated groups (Dunn’s, *P* = 0.0012) (**Figure 1**). These data demonstrate that local intranasal IFN-α administration synergises with systemic AZD1222 to elicit T-cell responses at the URT. Importantly, animals exhibited stable body weights and no signs of clinical distress throughout the experiment, indicating that this regimen was safe and well-tolerated.

**Figure 1:**
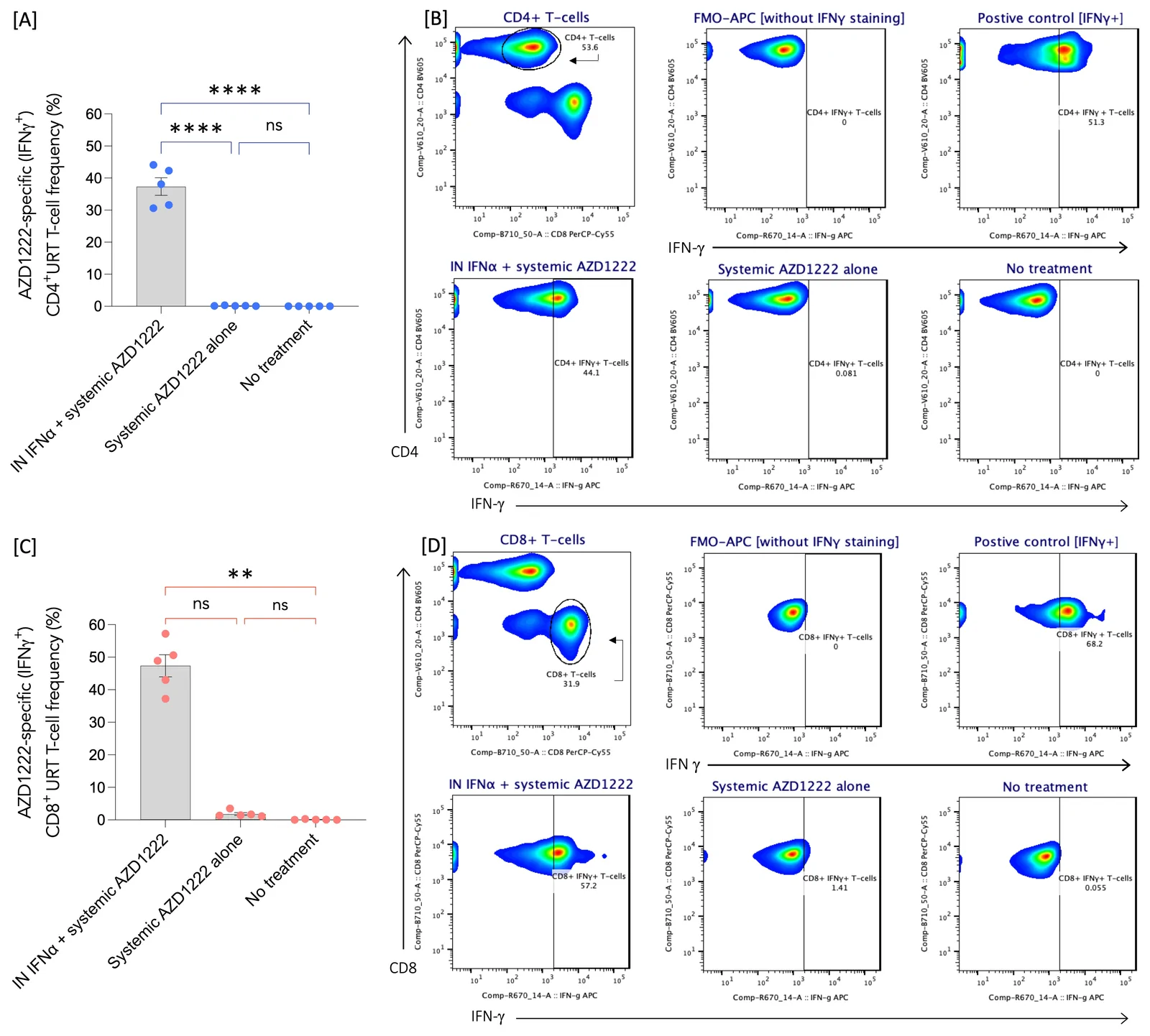
Intranasal IFN-α and systemic AZD1222 coadministration induced vaccine-specific CD4^+^ and CD8^+^ T-cell responses in the upper respiratory tract (URT). [A] IFN-γ expression in CD4^+^ T-cells was significantly higher in the intranasal IFN-α + systemic AZD1222 group compared to systemic AZD1222 alone, and also compared to the untreated group. Data were analysed using one-way ANOVA (****P < 0.0001) and *post-hoc* Tukey’s pairwise multiple comparison tests; ****P < 0.0001, ns: not significant. Data are shown as mean ± SEM with individual points representing individual mice; n=5 per experimental group. [B] Representative flow cytometry plots show (left to right, in row order) gated CD4^+^ T-cells; and CD4^+^IFN-γ^+^ T-cell frequencies in the fluorescence-minus-one (FMO) negative control (without IFN-γ-APC staining, which defines the background fluorescence threshold by using all antibodies except IFN-γ-APC to distinguish true IFN-γ-positive events from nonspecific signals), positive control, intranasal IFN-α + systemic AZD1222 group, systemic AZD1222 alone group, and no treatment group. CD4 expression is represented on the y-axis, and IFN-γ expression is represented along the x-axis. [C] IFN-γ expression in CD8^+^ T-cells was significantly higher in the intranasal IFN-α + systemic AZD1222 group compared to the untreated group. Data were analysed by applying Kruskal-Wallis (****P < 0.0001) and *post-hoc* Dunn’s pairwise multiple comparison tests; **P < 0.01; ns: not significant. Data are shown as mean ± SEM with individual points representing individual mice; n=5 per experimental group. [D] Representative flow cytometry plots show (left to right, in row order) gated CD8^+^ T-cells; and CD8^+^IFN-γ^+^ T-cell frequencies in FMO negative control, positive control, intranasal IFN-α + systemic AZD1222 group, systemic AZD1222 alone group, and no treatment group. CD8 expression is represented on the y-axis, and IFN-γ expression is represented along the x-axis.

### Intranasal IFN-α administration alone does not induce T-cell activation

In an additional control experiment, mice were administered intranasal IFN-α alone, without systemic AZD1222 coadministration, to determine whether IFN-α alone could induce URT T-cell activation. It was confirmed that administration of intranasal IFN-α alone is insufficient to trigger CD4^+^ or CD8^+^ T-cell activation responses in the absence of antigenic input from systemic vaccination (Kruskal-Wallis, *P* = 0.5205 and *P* = 0.8335, respectively, and Dunn’s *post-hoc* multiple comparison tests, *P* > 0.79) (**Figure 2**).

**Figure 2:**
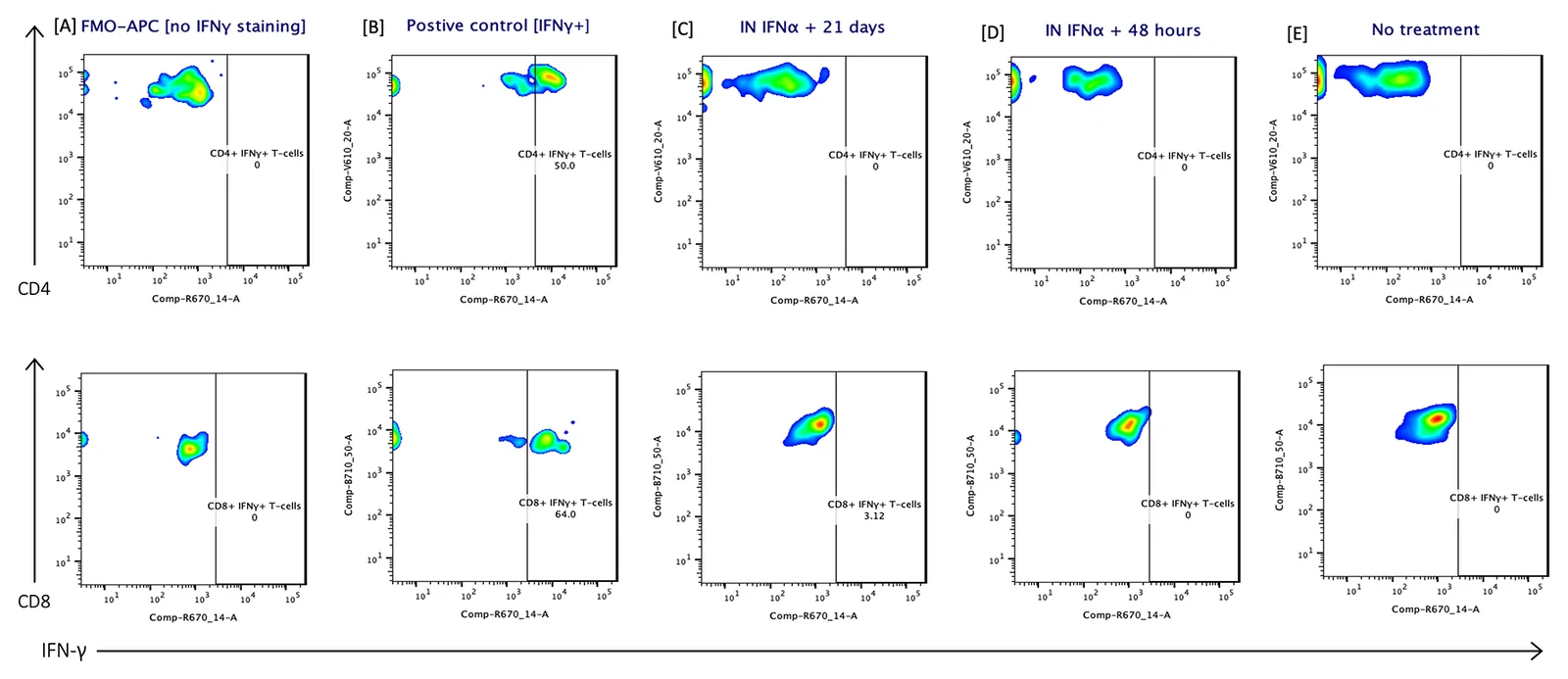
No CD4^+^ or CD8^+^ T-cell activation detected following intranasal IFN-α administration alone. Representative flow cytometric analysis for IFN-γ^+^CD4^+^ or IFN-γ^+^CD8^+^ T-cells (top and bottom row, respectively) at 48 hours post-IFN-α administration vs. 21 days post-IFN-α administration vs. no treatment; n=5 per experimental group. **(A)** FMO negative control, without IFN-γ–APC staining; **(B)** positive control; **(C)** intranasal IFN-α + 21 days; **(D)** intranasal IFN-α + 48 hours; **(E)** no treatment. CD4 and CD8 expression are represented on the y-axis, and IFN-γ expression is represented along the x-axis.

## Discussion

This preclinical proof-of-concept study was designed to assess whether a systemically delivered vaccine can achieve URT localisation of antigen-specific responses through concomitant exogenous nasal immunomodulation, and specifically in the absence of local antigen exposure. The exogenous nasal IFN-α signal in our adjunct strategy thus bypasses the presumed requirement for local antigen exposure, providing a paradigm-shifting method of directing a systemic vaccine-mediated response to the URT mucosa, where initial host infection and onward transmission occur.

Effective URT protection requires local immune responses. Systemic vaccination does not induce this URT signalling and therefore fails to direct antigen-specific responses to the nasal mucosa. In natural respiratory infection, type 1 interferons (IFN-1s; which include IFN-α), are produced at mucosal surfaces in response to viral antigenic presence and drive expression of interferon-stimulated genes (ISGs) that inhibit viral replication and establish a mucosal proinflammatory tissue environment supporting immune cell recruitment and memory development (14, 15). Intranasal IFN-α administration can replicate these effects even in the absence of local antigen encounter, by engaging IFNARs on epithelial and myeloid cells within the URT mucosa, and subsequently triggering a self-amplifying cascade of IFN-I production (16, 17). The resulting local inflammatory milieu promotes the expression of chemokines, cytokines and adhesion molecules to amplify inflammation, and upregulates endothelial adhesion molecule expression in local vascular beds to facilitate transendothelial lymphocyte migration into the tissue (14, 18–20). This chemotactic environment may thus enable the recruitment of vaccine-primed lymphocytes to the URT, even without local antigenic cues. Our intranasal adjunct strategy therefore provides a novel means of establishing URT mucosal immunity, by harnessing the early host innate response mechanism to respiratory infection, but crucially without dependence on local antigenic input in the URT.

Importantly, newly activated lymphocytes, such as those induced by systemic vaccination, exhibit a transiently heightened capacity to migrate to inflamed tissues, due to upregulation of homing receptors (integrins, selectins, chemokine receptors) that increase sensitivity to chemotactic cues and enable trafficking to distant sites expressing inflammatory signals (21–24); whilst previously generated effector lymphocytes downregulate their homing receptors by reverting to a resting state, with decreased migration dynamics towards inflamed sites (25, 26). As such, IFN-α-mediated inflammation in the nasal mucosa may preferentially recruit newly activated vaccine-induced T-cells, from the lower respiratory tract or the circulation, to the URT, and promote their retention through the establishment of tissue-resident memory cells (TRMs) (22). However, we acknowledge that the long-term fate of the URT T-cells induced by intranasal IFN-α administration, including whether they develop into TRMs or reenter the circulation, cannot be determined from the current study. Although the inflammatory mucosal context favours mucosal imprinting with URT TRM generation, definitive conclusions are not possible here. Further, it remains to be determined whether the URT T-cell responses arise from recruitment of vaccine-primed T-cells from the circulation or lower respiratory tract to the nasal mucosa, local T-cell activation and clonal expansion within nasopharyngeal/cervical lymphoid tissues following redirection of systemic vaccine antigen-bearing APCs to the URT, or a combination of both. Whilst non-specific bystander T-cell activation in response to local inflammatory cues could be considered a theoretical possibility, intranasal IFN-α administered in the absence of systemic vaccination did not generate detectable activated CD4^+^ or CD8^+^ T-cell responses in the URT.

Human intramuscular vaccination (administered in the upper arm deltoid muscle) drains into the axillary lymph nodes, facilitating systemic priming and mobilisation of immune cells capable of homing to the lower respiratory tract (5, 8). In contrast, murine intramuscular injections are administered in the hind limb, which drain to the inguinal and iliac lymph nodes to generate systemic responses that do not extend to the respiratory tract (27–29). However, intraperitoneal administration may represent an appropriate proxy for human systemic vaccination in the context of modelling systemic and lower respiratory immunity. This route provides broad systemic priming via drainage to mediastinal lymph nodes and spleen (30, 31), and can facilitate homing to the lower respiratory mucosa (32). Therefore, the intraperitoneal vaccination route employed here may better approximate the immunological dynamics of human intramuscular vaccination than the standard murine intramuscular route.

The adjunct strategy presented here may be widely applicable to multiple vaccines against diverse respiratory pathogens, for which the failure to induce effective URT mucosal immunity remains a major barrier to blocking infection and transmission. The proposed strategy additionally aligns with the WHO’s ‘Preparedness and Resilience for Emerging Threats’ initiative, which emphasises the need for adaptable, rapidly deployable pandemic responses (33). Notably, this approach could also reduce the need for frequent vaccine reformulation in response to evolving variants. Further, when vaccines prevent transmission, viral evolution towards highly transmissible variants is blocked; whilst high transmission rates in vaccinated populations can accelerate vaccine-resistant variant emergence (34, 35). Controlling infection and preventing transmission at the URT is therefore essential for breaking recurrent waves of infection and new vaccine-resistant variant emergence.

These findings establish exogenous intranasal IFN-α as an adjunct immunomodulatory signal capable of localising vaccine-specific responses to the URT, a site not directly accessed by systemic vaccination, through local instructive cues rather than reliance on intranasal antigen delivery. This proof-of-concept study therefore supports further development of targeted nasal immunomodulation with exogenous intranasal IFN-α as an impactful adjunct to systemic respiratory vaccination for the generation of URT mucosal immunity.

## Data Availability

The original contributions presented in the study are included in the article/**Supplementary Material**. Further inquiries can be directed to the corresponding author.
