# Supplementary material for: Exogenous IFN-α delivered as an intranasal adjunct to systemic vaccination directs vaccine-specific T-cell responses to the upper respiratory tract
Source: Front Immunol. 2026 Sep 18;17:1924673. doi: 10.3389/fimmu.2026.1924673 (PMC13631095; doi:10.3389/fimmu.2026.1924673)
Supplement: Supplementary file 1 [file DataSheet1.pdf]

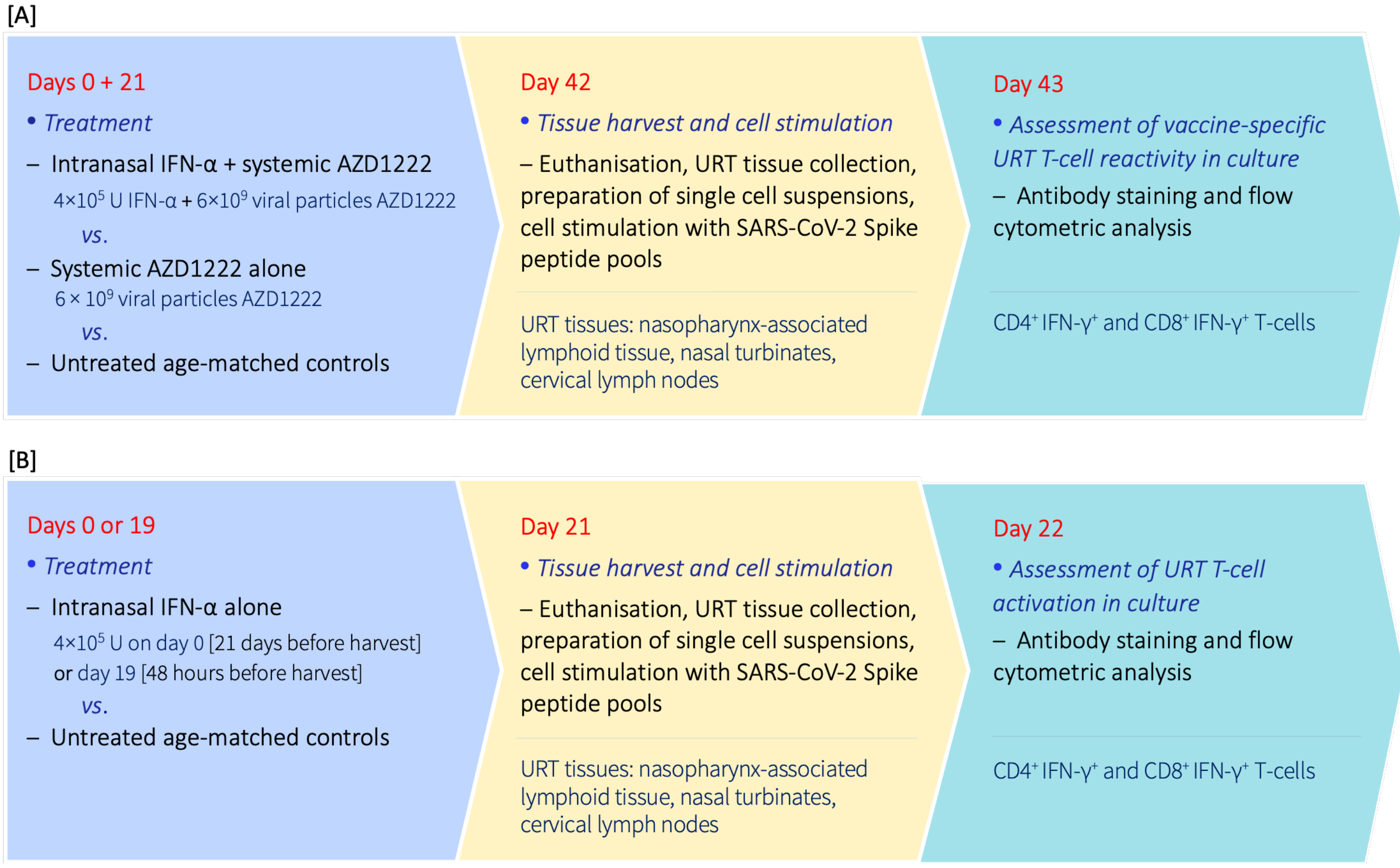

Supplementary figure 1. **Overview of experimental designs and timelines.** [A] Experimental design used to evaluate the induction of vaccine-specific upper respiratory tract (URT) T-cell responses following coadministration of intranasal IFN-α with systemic AZD1222. [B] Experimental design used to determine whether intranasal IFN-α alone induces URT T-cell activation.
