# Supplementary material for: Exogenous IFN-α delivered as an intranasal adjunct to systemic vaccination directs vaccine-specific T-cell responses to the upper respiratory tract
Source: Front Immunol. 2026 Sep 18;17:1924673. doi: 10.3389/fimmu.2026.1924673 (PMC13631095; doi:10.3389/fimmu.2026.1924673)
Supplement: Supplementary file 2 [file DataSheet2.pdf]

Raw data, $CD4^+IFN\gamma^+$ T-cells

Intranasal IFNα + systemic AZD1222	Systemic AZD1222 alone	No treatment
42.3	0.17	0
44.1	0.081	0.012
31.6	0.11	0
38.1	0.31	0.022
30.6	0.11	0.01

Shapiro-Wilk test for normal distribution of $CD4^+IFN\gamma^+$ T-cells

	Intranasal IFNα + systemic AZD1222	Systemic AZD1222 alone	No treatment
W	0.8946	0.8228	0.9005
P value	0.3808	0.1226	0.4127
Passed normality test (alpha=0.05)?	Yes	Yes	Yes
P value summary	ns	ns	ns

Ordinary one-way ANOVA of $CD4^+IFN\gamma^+$ T-cells

Table analysed	$CD4^+IFN\gamma^+$ T-cells
Distribution assumption	Normal (Gaussian)
F	186.0
P value	<0.0001
P value summary	****
Significant difference among means (P<0.05)?	Yes
R squared	0.9687

Tukey’s multiple comparisons test for $CD4^+IFN\gamma^+$ T-cells

Number of families	1
Number of comparisons per family	3
Alpha	0.05

Tukey's multiple comparisons test	Mean diff.	95.00% CI of diff.	Below threshold?	Summary	Adjusted P value
IN IFNα + systemic AZD1222 vs. systemic AZD1222 alone	37.18	31.23 to 43.14	Yes	****	<0.0001
IN IFNα + systemic AZD1222 vs. No treatment	37.33	31.38 to 43.28	Yes	****	<0.0001
Systemic AZD1222 alone vs. No treatment	0.1474	-5.804 to 6.099	No	ns	0.9976

Test details	Mean 1	Mean 2	Mean diff.	SE of diff.	n1	n2	q	DF
IN IFNα + systemic AZD1222 vs. Systemic AZD1222 alone	37.34	0.1562	37.18	2.231	5	5	23.57	12
IN IFNα + systemic AZD1222 vs. No treatment	37.34	0.008800	37.33	2.231	5	5	23.67	12
Systemic AZD1222 alone vs. No treatment	0.1562	0.008800	0.1474	2.231	5	5	0.09345	12

Raw data, CD8$^+$IFNγ$^+$ T-cells

Intranasal IFNα + systemic AZD1222	Systemic AZD1222 alone	No treatment
50.6	1.32	0.29
57.2	1.41	0.024
43	1.11	0.088
48.9	3.48	0.025
37.2	1.68	0.055

Shapiro-Wilk test for normal distribution of CD8$^+$IFNγ$^+$ T-cells

	Intranasal IFNα + systemic AZD1222	Systemic AZD1222 alone	No treatment
W	0.9866	0.7476	0.7431
P value	0.9667	0.0282	0.0257
Passed normality test (alpha=0.05)?	Yes	No	No
P value summary	ns	*	*

Kruskal-Wallis test of CD8$^+$IFNγ$^+$ T-cells

Table analysed	CD8$^+$IFNγ$^+$ T-cells
Distribution assumption	No assumption
P value	<0.0001
Exact or appropriate P value?	Exact
P value summary	****
Do the medians vary significantly (P<0.05)?	Yes
Number of groups	3
Kruskal-Wallis	12.50

Dunn's multiple comparisons test for CD8$^+$IFNγ$^+$ T-cells

Number of families	1
Number of comparisons per family	3
Alpha	0.05

Dunn's multiple comparisons test	Mean rank diff.	Significant?	Summary	Adjusted P value
IN IFNα + systemic AZD1222 vs. Systemic AZD1222 alone	5.00	No	ns	0.2313
IN IFNα + systemic AZD1222 vs. No treatment	10.00	Yes	**	0.0012
No treatment	5.00	No	ns	0.2313

Test details	Mean rank 1	Mean rank 2	Mean rank diff.	n1	n2	Z
IN IFNα + systemic AZD1222 vs. Systemic AZD1222 alone	13.00	8.00	5.00	5	5	1.768
IN IFNα + systemic AZD1222 vs. No treatment	13.00	3.00	10.00	5	5	3.536
No treatment	8.00	3.00	5.00	5	5	1.768

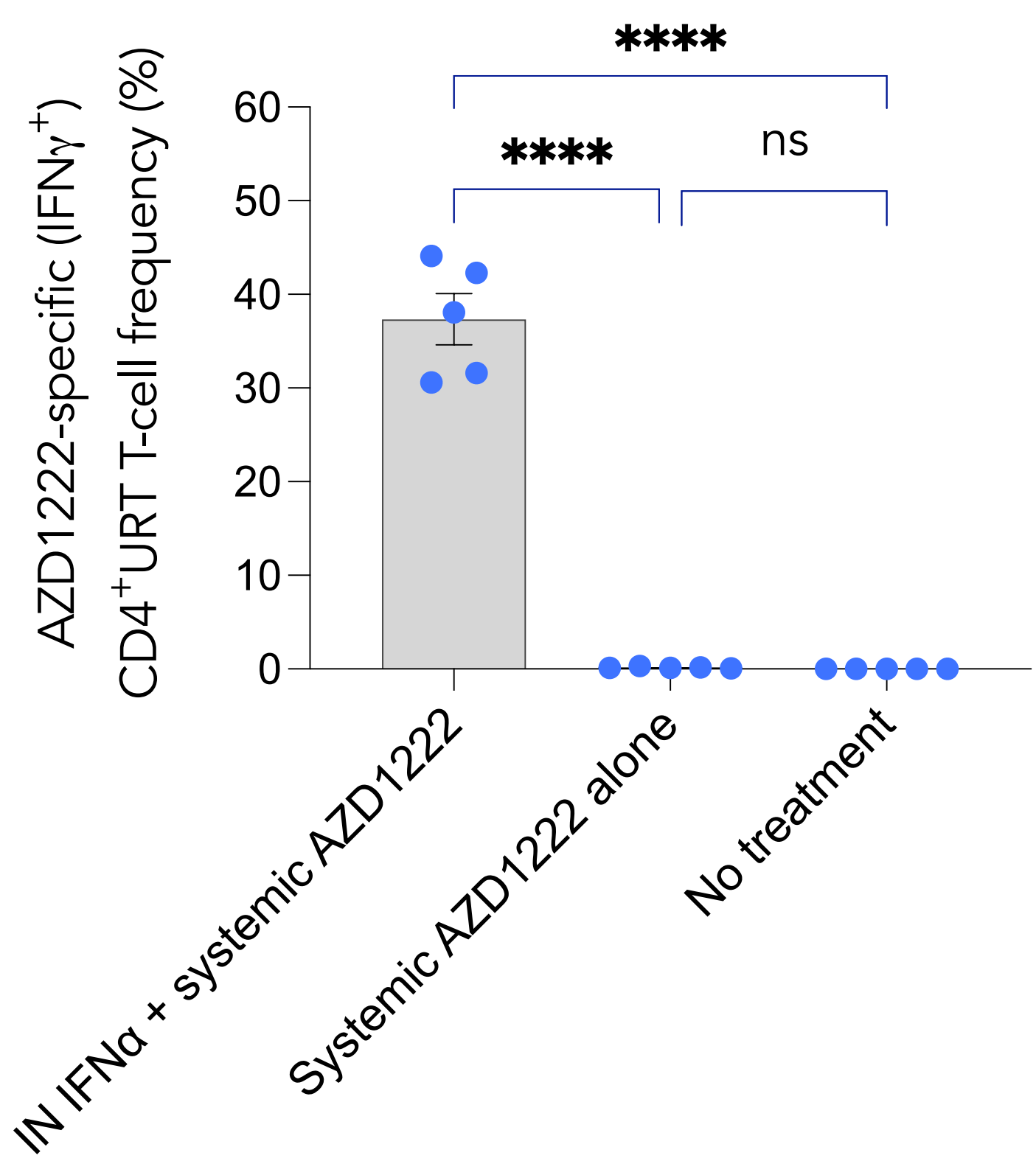
****
****
ns
60
50
40
30
20
10
0
AZD1222-specific (IFNγ+)
CD4+URT T-cell frequency (%)
IN IFNα + systemic AZD1222
Systemic AZD1222 alone
No treatment

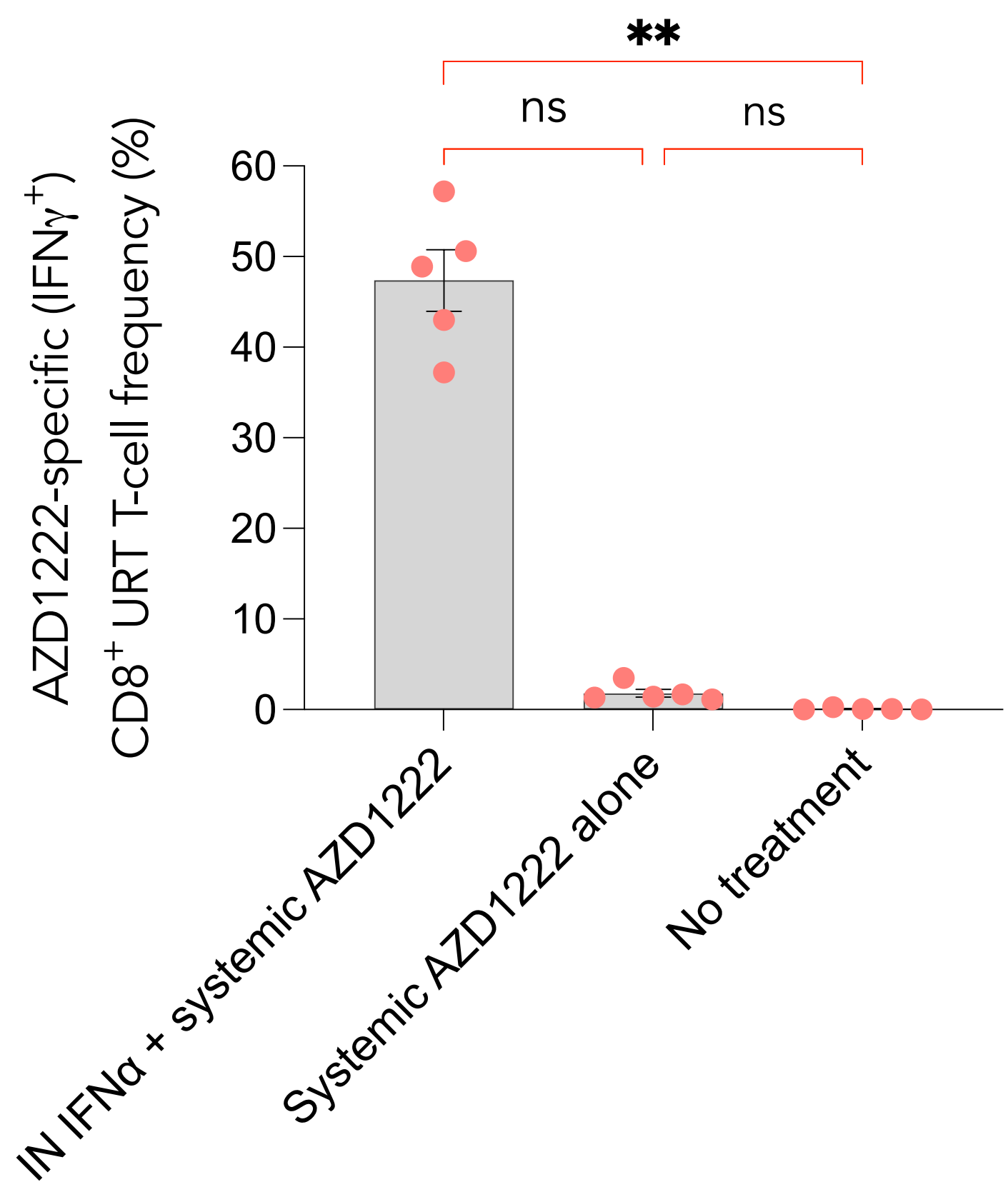
**
ns
ns
60
50
40
30
20
10
0
AZD1222-specific (IFNγ+)
CD8+ URT T-cell frequency (%)
IN IFNα + systemic AZD1222
Systemic AZD1222 alone
No treatment

Raw data, control experiment CD4$^+$IFNγ$^+$ T-cells

Intranasal IFNα + 21 days	Intranasal IFNα + 48 hours	No treatment
0	0.42	0
0	0	0
0	1.04	0
1.92	0	0
2.38	1.82	0.47

Shapiro-Wilk test for normal distribution of control experiment CD4$^+$IFNγ$^+$ T-cells

	Intranasal IFNα + 21 days	Intranasal IFNα + 48 hours	No treatment
W	0.7400	0.8821	0.5522
P value	0.0240	0.3189	0.0001
Passed normality test (alpha=0.05)?	No	Yes	No
P value summary	*	ns	***

Kruskal-Wallis test of control experiment CD4$^+$IFNγ$^+$ T-cells

Table analysed	Control experiment CD4$^+$IFNγ$^+$ T-cells
Distribution assumption	No assumption
P value	0.5205
Exact or appropriate P value?	Exact
P value summary	ns
Do the medians vary significantly (P<0.05)?	No
Number of groups	3
Kruskal-Wallis	1.553

Dunn's multiple comparisons test for control experiment CD4$^+$IFNγ$^+$ T-cells

Number of families	1
Number of comparisons per family	3
Alpha	0.05

Dunn's multiple comparisons test	Mean rank diff.	Significant?	Summary	Adjusted P value
IN IFNα + 21 days	-0.2000	No	ns	>0.9999
IN IFNα + 48 hours	2.600	No	ns	0.8991
No treatment	2.800	No	ns	0.7922

Test details	Mean rank 1	Mean rank 2	Mean rank diff.	n1	n2	Z
IN IFNα + 21 days	8.800	9.000	-0.2000	5	5	0.07977
IN IFNα + 48 hours	8.800	6.200	2.600	5	5	1.037
No treatment	9.000	6.200	2.800	5	5	1.117

Raw data, control experiment CD8$^+$IFNγ$^+$ T-cells

Intranasal IFNα + 21 days	Intranasal IFNα + 48 hours	No treatment
2.82	1.04	0
0	0	0
0	0	0.42
0.5	0	0.34
0	0	0

Shapiro-Wilk test for normal distribution of control experiment CD8$^+$IFNγ$^+$ T-cells

	Intranasal IFNα + 21 days	Intranasal IFNα + 48 hours	No treatment
W	0.6635	0.5522	0.7393
P value	0.0038	0.0001	0.0236
Passed normality test (alpha=0.05)?	No	No	No
P value summary	**	***	*

Kruskal-Wallis test of control experiment CD8$^+$IFNγ$^+$ T-cells

Table analysed	Control experiment CD8$^+$IFNγ$^+$ T-cells
Distribution assumption	No assumption
P value	0.8335
Exact or appropriate P value?	Exact
P value summary	ns
Do the medians vary significantly (P < 0.05)?	No
Number of groups	3
Kruskal-Wallis	1.5175

Dunn's multiple comparisons test for control experiment CD8$^+$IFNγ$^+$ T-cells

Number of families	1
Number of comparisons per family	3
Alpha	0.05

Dunn's multiple comparisons test	Mean rank diff.	Significant?	Summary	Adjusted P value
IN IFNα + 21 days	1.700	No	ns	>0.9999
IN IFNα + 48 hours	1.000	No	ns	>0.9999
No treatment	-0.7000	No	ns	>0.9999

Test details	Mean rank 1	Mean rank 2	Mean rank diff.	n1	n2	Z
IN IFNα + 21 days	8.900	7.200	1.700	5	5	0.7156
IN IFNα + 48 hours	8.900	7.900	1.000	5	5	0.4210
No treatment	7.200	7.900	-0.7000	5	5	0.2947

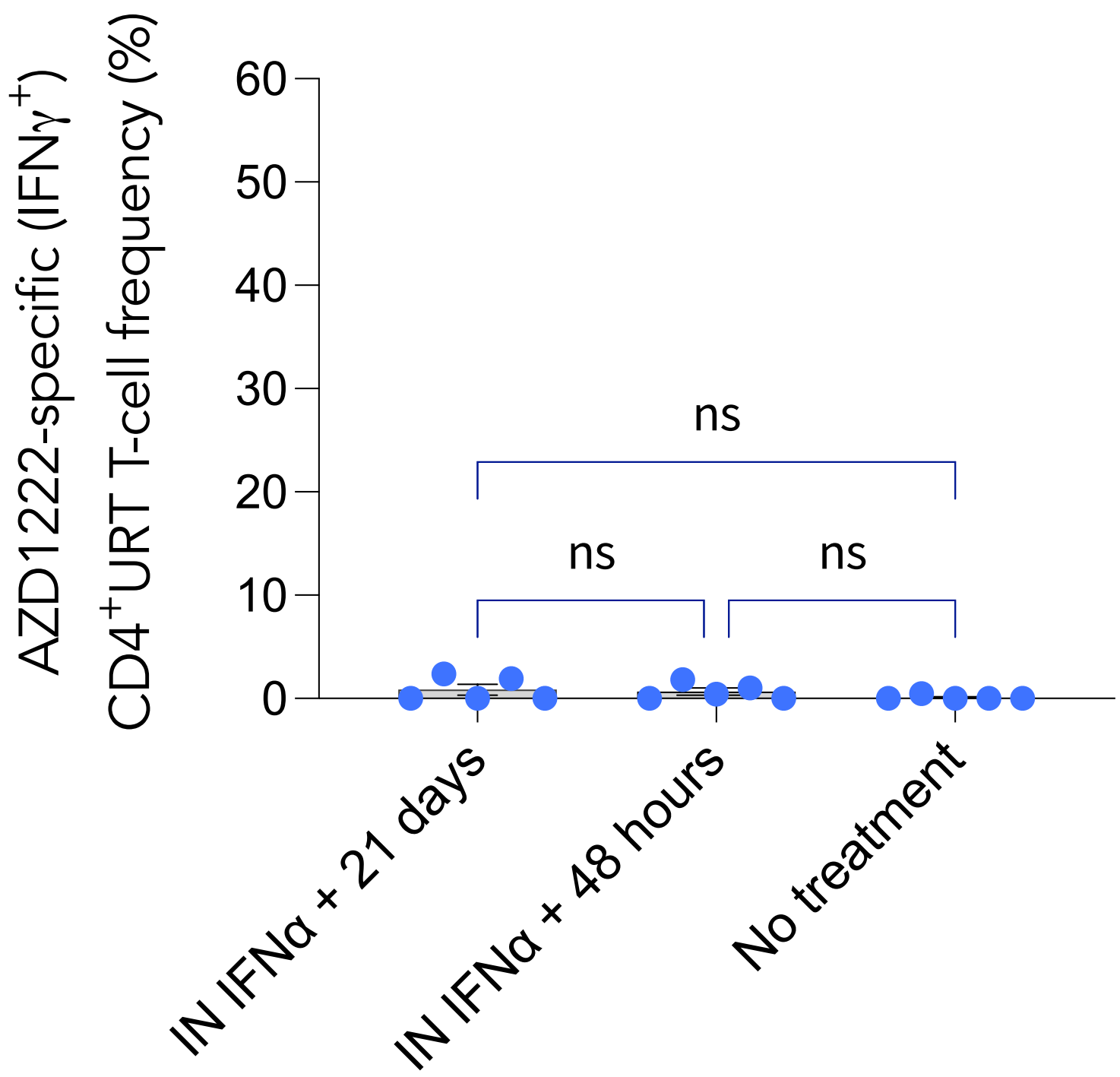
AZD1222-specific (IFNγ+)
CD4+URT T-cell frequency (%)
60
50
40
30
20
10
0
ns
ns
ns
IN IFNα + 21 days
IN IFNα + 48 hours
No treatment

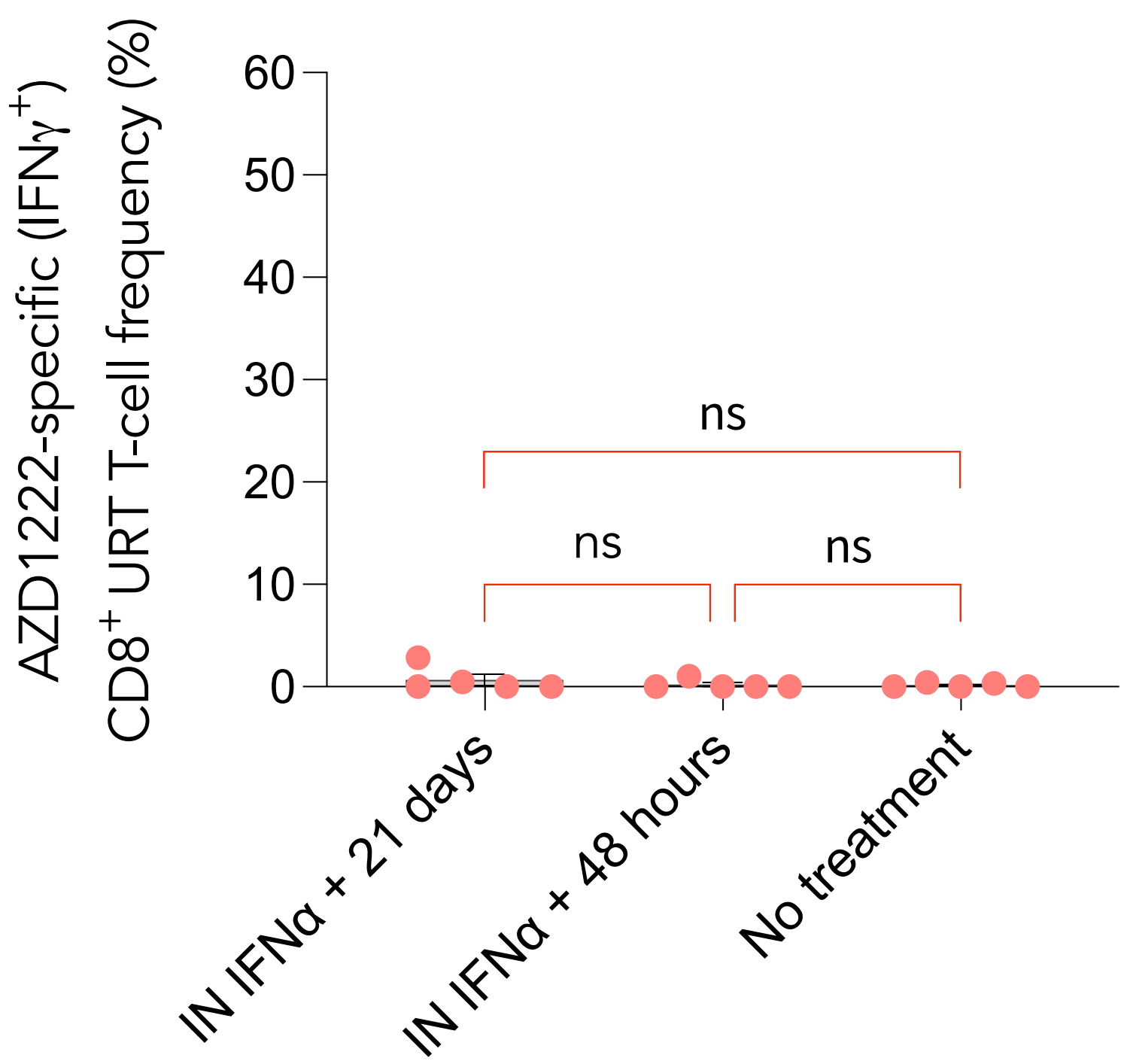
AZD1222-specific (IFNγ+)
CD8+ URT T-cell frequency (%)
60
50
40
30
20
10
0
ns
ns
ns
IN IFNα + 21 days
IN IFNα + 48 hours
No treatment
